# ^13^C-glucose breath tests: a non-invasive method for detecting early clinical manifestations of exogenous glucose metabolism in type 2 diabetic patients

**DOI:** 10.1007/s00592-018-1276-y

**Published:** 2018-12-28

**Authors:** Ikutaka Takemoto, Naoyuki Kawagoe, Sho Kijima, Yosuke Sasaki, Toshiyasu Watanabe, Yoshihisa Urita

**Affiliations:** Department of General Medicine and Emergency Care, School of Medicine, Toho University, 6-11-1 Omorinishi, Ota-ku, Tokyo, 143-8541 Japan

**Keywords:** Combined [1-^13^C], [2-^13^C], [3-^13^C]glucose breath test, Metabolic fate, Diabetes mellitus, Glucose uptake, Glucose oxidation, Gluconeogenesis

## Abstract

**Aims:**

^13^C-glucose breath tests are reported as an alternative non-invasive method to evaluate glucose metabolism. However, the metabolic results differ based on the site of the carbon atom in the glucose. The aim of this study was to evaluate changes in the metabolism of carbon atoms contained in glucose in patients with diabetes using [1, 2, 3-^13^C]glucose breath tests.

**Methods:**

Sixteen healthy participants and 20 diabetic patients were enrolled in the study. Three types of breath tests, [1-^13^C], [2-^13^C], and [3-^13^C]glucose breath tests, were performed after an overnight fast. Breath samples were taken at baseline and at 10-min intervals over 150 min, and ^13^CO_2_ excretion curves were expressed using non-dispersive infrared isotope spectrometry.

**Results:**

^13^CO_2_ levels increased more rapidly, and the peak value of ^13^CO_2_ (*C*_max_) was highest after the administration of [3-^13^C]glucose followed by [2-^13^C] and [1-^13^C]glucose in controls. Delayed ^13^CO_2_ excretion and a low area under the curve through 150 min (AUC_150_) were obtained in diabetic patients. The group with severe diabetes had a significantly lower *C*_max_ and AUC_150_ in the [1-^13^C]glucose breath test.

**Conclusions:**

The [1-^13^C]glucose breath test, which has been used to evaluate glucose metabolism, is suitable for patients with late-stage diabetes, whereas the [2-^13^C]glucose breath test is ideal in the early stages. Although the [3-^13^C]glucose breath test is theoretically useful for evaluating the uptake of glucose and the anaerobic glycolysis system, it can be used in practice to distinguish reduced uptake from impaired oxidation of glucose in combination with the other two tests.

## Introduction

Although increased plasma glucose levels directly reflect the total amount of glucose ingested, postprandial levels can vary even after ingestion of food with the same amount of energy. Postprandial plasma glucose levels are regulated by complicated processes including gastric emptying, digestion, absorption, insulin secretion and resistance, and glucose uptake by cells. Carbohydrates, which are easily digested and promptly absorbed, result in high plasma glucose concentrations and increased insulin demand and may, therefore, contribute to an increased risk of type 2 diabetes [1]. Insulin resistance is closely associated with the progression of type 2 diabetes, which should be diagnosed at an asymptomatic stage to prevent fatal complications such as ischemic heart disease, stroke, and renal failure [2]. It is, however, quite difficult for asymptomatic patients to go to a hospital for a medical check-up. One reason is the fear of the needles used to take blood samples to measure plasma glucose levels. Alternative non-invasive methods that do not cause anxiety are thus desirable to encourage patients to go in for tests, to estimate their risk for diabetes.

Recently, ^13^C-glucose breath tests have been reported as an alternative method of testing for insulin resistance [3–5]. Breath tests using ^13^C substrates are based on the principle that ^13^CO_2_ in the exhaled breath can be measured as a metabolic tracer that originates from the following process: absorption from the small intestine, metabolism, and oxidation, and ^13^CO_2_ excretion from the respiratory system. However, different protocols for ^13^C-glucose breath tests have been reported. In addition, the carbon atoms in glucose have different metabolic fates, depending on whether the glucose is metabolized via glycolysis, gluconeogenesis, or the pentose phosphate pathway, resulting in the formation of tricarboxylic acid (TCA) cycle intermediates, lactate, alanine, glutamate, and/or γ-aminobutyric acid (GABA), and CO_2_ [6]. Based on the biochemical characteristics of glucose metabolism, we have previously reported an animal study using [1, 2, 3-^13^C]glucose breath tests. In that study, we concluded that the utilization of [2-^13^C]glucose was suppressed in the early stage of prediabetes and that the metabolism of [3-^13^C]glucose was enhanced just before the onset of diabetes [7]. Thus, it is possible that these three types of ^13^C-glucose breath tests could be an alternative and non-invasive method for detecting the early stage of type 2 diabetes.

The aim of this study was to evaluate changes in the metabolism of the carbon atoms that make up glucose in diabetic patients using [1, 2, 3-^13^C]glucose breath tests.

## Materials and methods

### Participants

This experimental study was conducted at the Toho University Omori Medical Center and Social Insurance Kamata General Hospital. The study protocol was approved by the ethics committees of both institutions. Sixteen healthy volunteers aged between 20 and 23 years (mean age = 21.5 ± 1.0 years, male:female = 5:8) and 20 diabetic patients aged between 37 and 82 years (mean age = 58.4 ± 14.4 years, male:female = 15:5) were enrolled in the study. As shown in Table 1, the body weight and body mass index (BMI) were significantly greater in the diabetic patients than in the controls.

**Table 1:** Characteristics of diabetic and control groups

	Diabetes (*n* = 20)	Control (*n* = 16)	*P*
Age (years)	58.4 ± 14.4	21.5 ± 1.0	< 0.001
Gender (m/f)	15/5	7/9	NS
Height (cm)	162.7 ± 8.5	164.4 ± 10.1	NS
Weight (kg)	65.2 ± 12.1	56.1 ± 9.0	0.018
BMI (kg/m^2^)	24.6 ± 4.0	20.8 ± 2.7	0.002
HbA1c (%)	9.2 ± 2.2	Not examined	

*NS* not significant

Diabetic patients were categorized based on their glycated hemoglobin (HbA1c) levels at the time of enrolment as severe (HbA1c ≥ 10.0%) or non-severe (HbA1c < 10.0%). There was no parameter with a significant difference between the two groups except for HbA1c (Table 2).

**Table 2:** Characteristics of the two diabetic groups classified based on HbA1c

	HbA1c ≥ 10% (*n* = 7)	HbA1c < 10% (*n* = 13)	*P*
Age (years)	58.7 ± 16.4	58.2 ± 14.0	NS
Gender (m/f)	5/2	10/3	NS
Height (cm)	161.8 ± 9.3	163.1 ± 8.4	NS
Weight (kg)	64.1 ± 13.2	65.8 ± 11.9	NS
BMI (kg/m^2^)	24.5 ± 4.9	24.6 ± 3.6	NS
HbA1c (%)	11.6 ± 1.5	7.9 ± 1.0	< 0.001

*NS* not significant

Informed consent was obtained from all the participants. Participants who had a previous history of partial gastrectomy were excluded from the study. The participants had not taken any medication known to alter plasma glucose levels 6 months prior to the study.

### Breath test

Three types of breath tests, [1-^13^C], [2-^13^C], and [3-^13^C]glucose breath tests, were performed in the sitting position after an overnight fast. The patients received 100 mL of water containing 100 mg of ^13^C-glucose (Cambridge Isotope Laboratories, Inc., Tewksbury, MA, USA). Breath samples were taken at baseline and at 10-min intervals over 150 min, and ^13^CO_2_ levels were measured. The ^13^C levels were estimated as the ^13^CO_2_/^12^CO_2_ isotope ratio and were expressed as delta over baseline per mil (Δ‰) using non-dispersive infrared isotope spectrometry (POCone; Otsuka Electrics Co, Ltd., Hirakata-shi, Japan).

The results were converted to the percentage of ^13^CO_2_ recovered in the breath per hour (%dose/h) based on the body surface area (BSA) and the assumed CO_2_ production (${\dot {V}_{{\text{C}}{{\text{O}}_2}}}$) as follows [8]:

$$\% {\text{dose}}/h=\Delta \permille \times {\dot {V}_{{\text{C}}{{\text{O}}_2}}} \times 0.01123 \times 10/(A \times {\text{APE}}/{\text{MW}}),$$

where the molecular weight (MW) is 180, ${\dot {V}_{{\text{C}}{{\text{O}}_2}}}$ is 300 BSA mmol/h, BSA is 0.024265 × weight^0.5378^ × height^0.3964^ m^2^, dose (*A*) is 100 mg, and atom% excess (APE) is 99 atom%. The maximum concentration (*C*_max_, %dose/h), the time taken to reach the maximum concentration (*T*_max_, minutes), and the area under the curve over 150 min (AUC_150_, %dose/h min) were calculated. *C*_max_ and the AUC_150_ reflect the absorption of the labeled substrate.

### Statistical analysis

Results are reported as the mean ± standard deviation unless otherwise indicated. Student’s *t* test was used to examine between-group differences in AUC_150_, and *C*_max_ and *P* < 0.05 was regarded as significant. All statistical analyses were performed with Easy R (EZR; Saitama Medical Center, Jichi Medical University, Saitama, Japan), a graphical user interface for R (The R Foundation for Statistical Computing, version 3.2.2) [9].

## Results

**Fig. 1:**
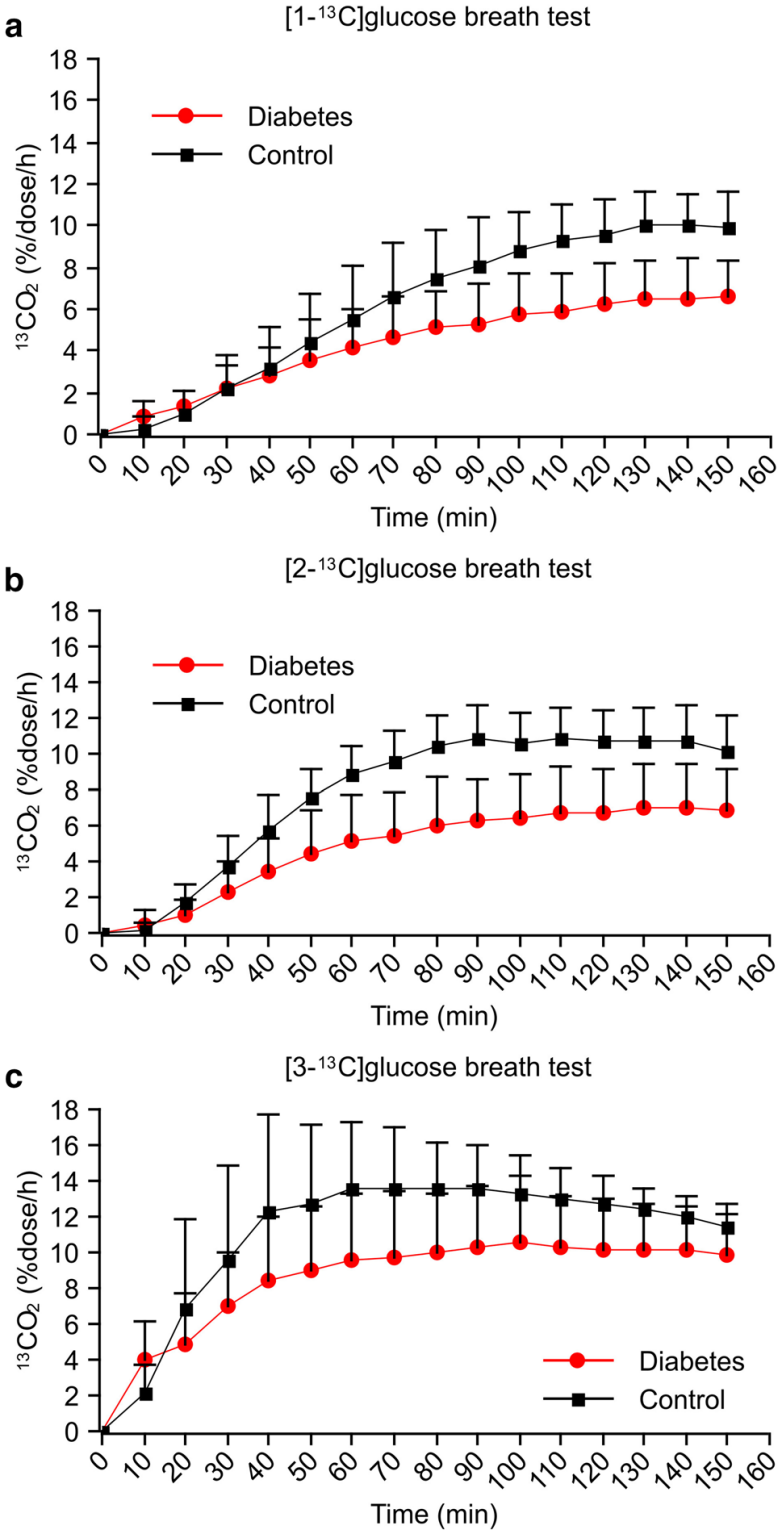
^13^CO_2_ excretion curves. **a** Comparison of orally administered [1-^13^C]glucose in the diabetic and control groups. **b** Comparison of orally administered [2-^13^C]glucose in the diabetic and control groups. **c** Comparison of orally administered [3-^13^C]glucose in the diabetic and control groups

The average ^13^CO_2_ excretion levels at each sampling point after oral administration of the ^13^C-glucose (100 mg) are shown in Fig. 1a–c. All of the ^13^CO_2_ concentrations except at the 10-min time point were lower in diabetic patients than in controls for all the three types of ^13^C-glucose breath tests. After administering the three types of ^13^C-glucose, the increase in breath ^13^CO_2_ excretion was delayed in diabetic patients compared with controls. *C*_max_ was 11.3 ± 1.6%dose/h, 7.5 ± 2.5%dose/h, and 7.0 ± 1.9%dose/h in the diabetic patients after ingestion of the [3-^13^C], [2-^13^C], and [1-^13^C]glucose solutions, respectively. The *C*_max_ values for all the breath tests were significantly lower in diabetic patients than in the controls (Table 3). Similarly, no significant difference in AUC_150_ was observed between the severe and non-severe diabetes groups in the [3-^13^C] or [2-^13^C]glucose breath test, whereas a significant difference was observed between diabetic patients and controls for all breath tests (Table 4).

**Table 3:** *C*_max_ values of all breath tests in the diabetic and control groups

	Diabetes (*n* = 20)	Control (*n* = 16)	*P*
[1-^13^C]glucose	7.0 ± 1.9	10.4 ± 1.6	< 0.001
[2-^13^C]glucose	7.5 ± 2.5	11.3 ± 1.8	< 0.001
[3-^13^C]glucose	11.3 ± 3.5	14.8 ± 3.0	0.007

Data are represented as mean (%dose/h) ± SD

*NS* not significant

**Table 4:** AUC_150_ values of all breath tests in diabetic and control groups

	Diabetes (*n* = 20)	Control (*n* = 18)	*P*
[1-^13^C]glucose	642 ± 210	914 ± 226	< 0.001
[2-^13^C]glucose	717 ± 281	1174 ± 178	< 0.001
[3-^13^C]glucose	1289 ± 409	1668 ± 400	0.016

Data are represented as mean [(%dose/h) min] ± SD

*NS* not significant

**Fig. 2:**
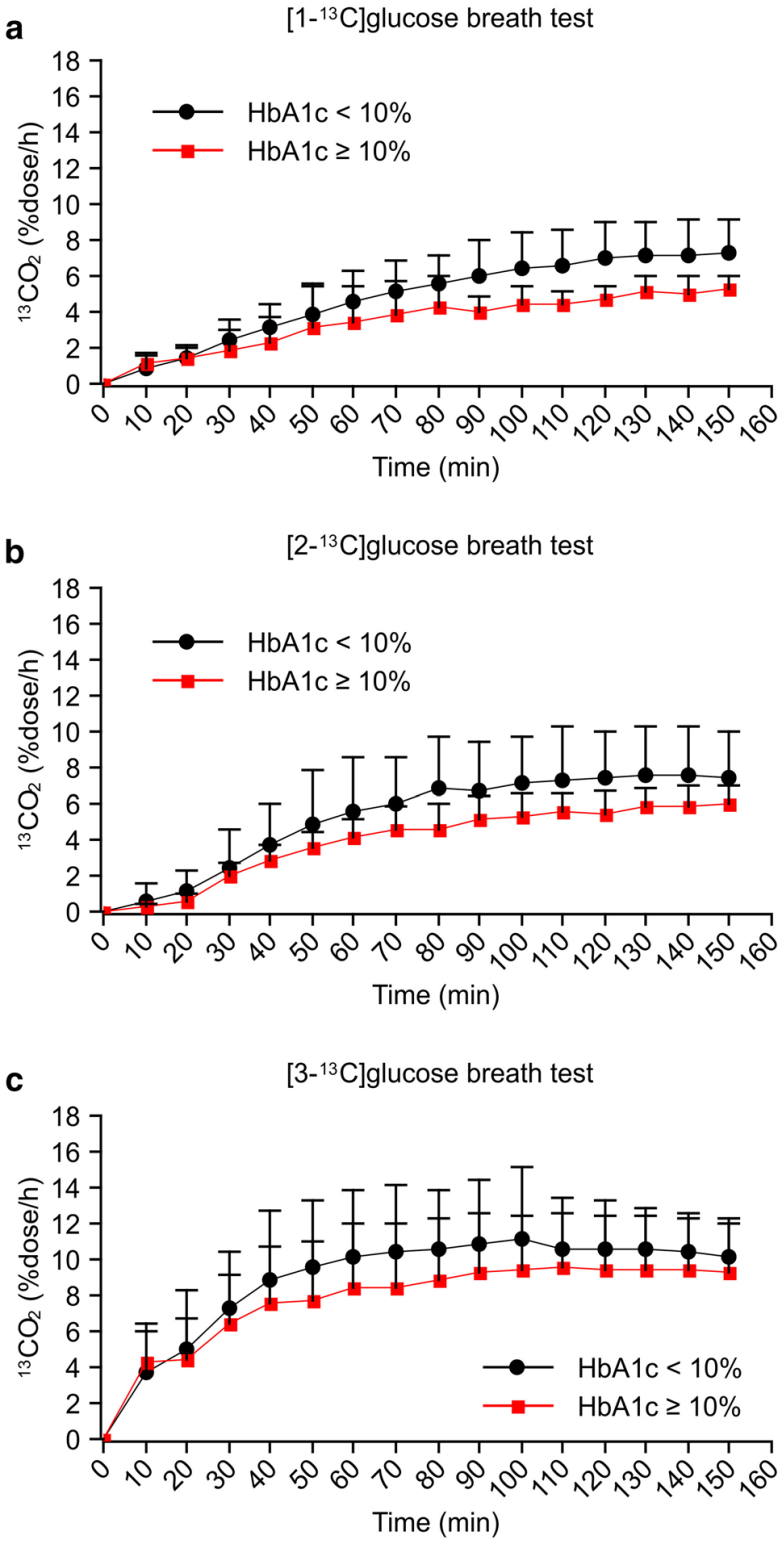
^13^CO_2_ excretion curves. **a** Comparison of orally administered [1-^13^C]glucose in the two diabetic groups classified based on HbA1c. **b** Comparison of orally administered [2-^13^C]glucose in the two diabetic groups classified based on HbA1c. **c** Comparison of orally administered [3-^13^C]glucose in the two diabetic groups classified based on HbA1c

Among diabetic patients, Fig. 2a–c shows the ^13^CO_2_ excretion curves for the severe and non-severe diabetes groups after the oral administration of [1-^13^C], [2-^13^C], and [3-^13^C]glucose. The severe diabetes group had a significantly lower *C*_max_ value on the [1-^13^C]glucose breath test compared with the non-severe group (Table 5). There was a tendency for patients with severe diabetes to have lower *C*_max_ values on both the [3-^13^C] and [2-^13^C]glucose breath tests, but the differences were not statistically significant. In contrast with the [2-^13^C]glucose and [3-^13^C]glucose breath tests, there was a significant difference in the AUC_150_ between severe and non-severe diabetes patients after the oral administration of [1-^13^C]glucose (Table 6).

**Table 5:** *C*_max_ values of all breath tests in the two diabetic groups classified based on HbA1c

	HbA1c ≥ 10% (*n* = 7)	HbA1c < 10% (*n* = 13)	*P*
[1-^13^C]glucose	5.8 ± 1.3	7.6 ± 1.9	0.039
[2-^13^C]glucose	6.2 ± 1.0	8.2 ± 2.8	0.087
[3-^13^C]glucose	10.1 ± 2.8	11.9 ± 3.7	0.295

Data are represented as mean (%dose/h) ± SD

*NS* not significant

**Table 6:** AUC_150_ values of all breath tests in the two diabetic groups classified based on HbA1c

	HbA1c ≥ 10% (*n* = 7)	HbA1c < 10% (*n* = 13)	*P*
[1-^13^C]glucose	517 ± 148	708 ± 212	0.049
[2-^13^C]glucose	590 ± 124	785 ± 320	0.142
[3-^13^C]glucose	1175 ± 393	1350 ± 420	0.378

Data are represented as mean [(%dose/h) min] ± SD

*NS* not significant

**Fig. 3:**
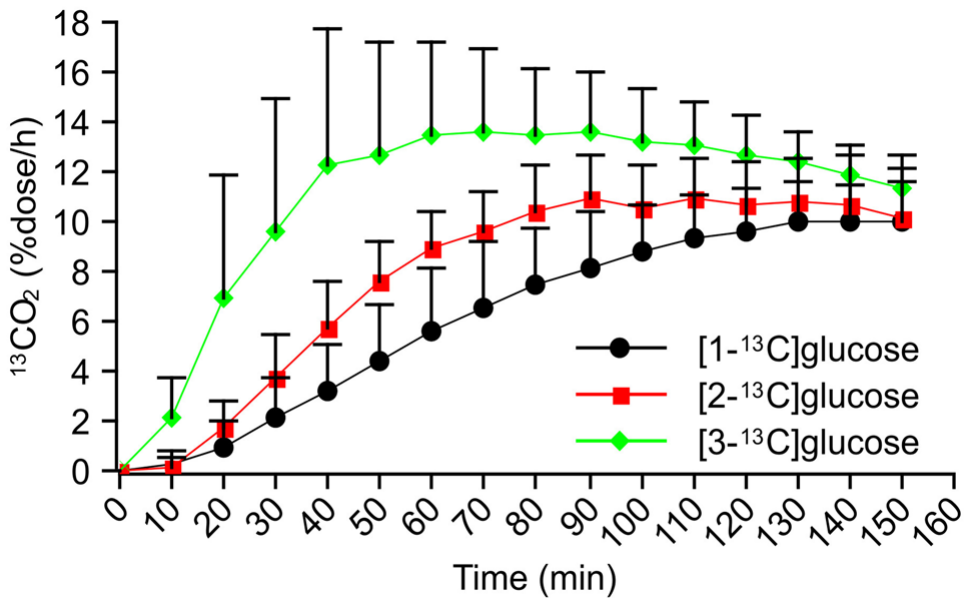
^13^CO_2_ excretion curves after oral administration of three types of labeled glucose in controls

**Fig. 4:**
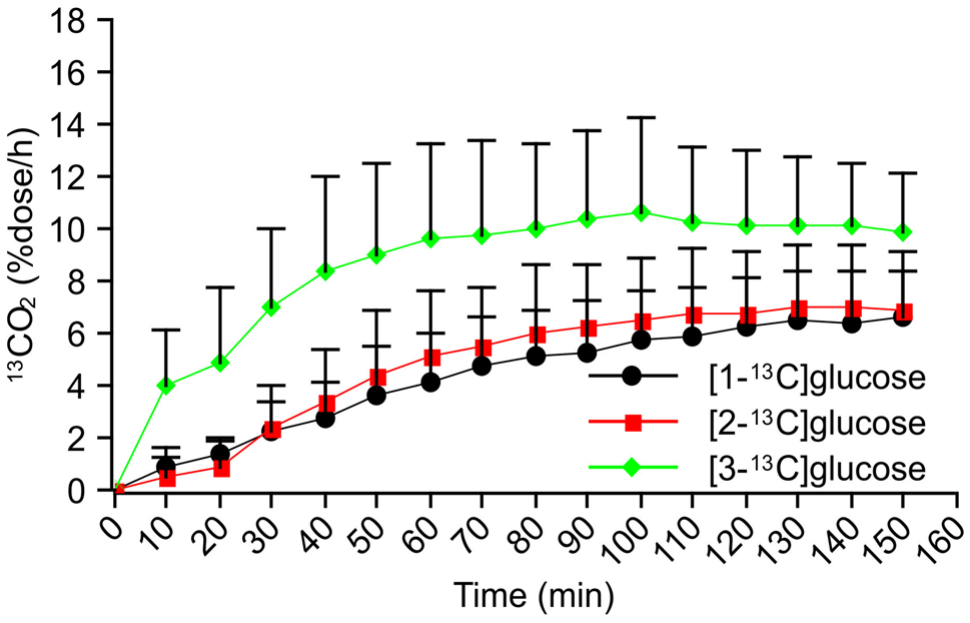
^13^CO_2_ excretion curves after oral administration of three types of labeled glucose in diabetic patients

When comparing the ^13^CO_2_ excretion curves for the three types of ^13^C-glucose breath tests in the control group, the ^13^CO_2_ levels increased more rapidly, and *C*_max_ values were higher after the administration of [3-^13^C]glucose than after the administration of [2-^13^C] and [1-^13^C]glucose (Fig. 3). For the diabetic patients, although ^13^CO_2_ levels increased more rapidly and had higher peak levels in the [3-^13^C]glucose breath test, the differences in the ^13^CO_2_ excretion curves between the [2-^13^C] and [1-^13^C]glucose breath tests were markedly reduced, and the two curves were quite similar (Fig. 4).

**Fig. 5:**
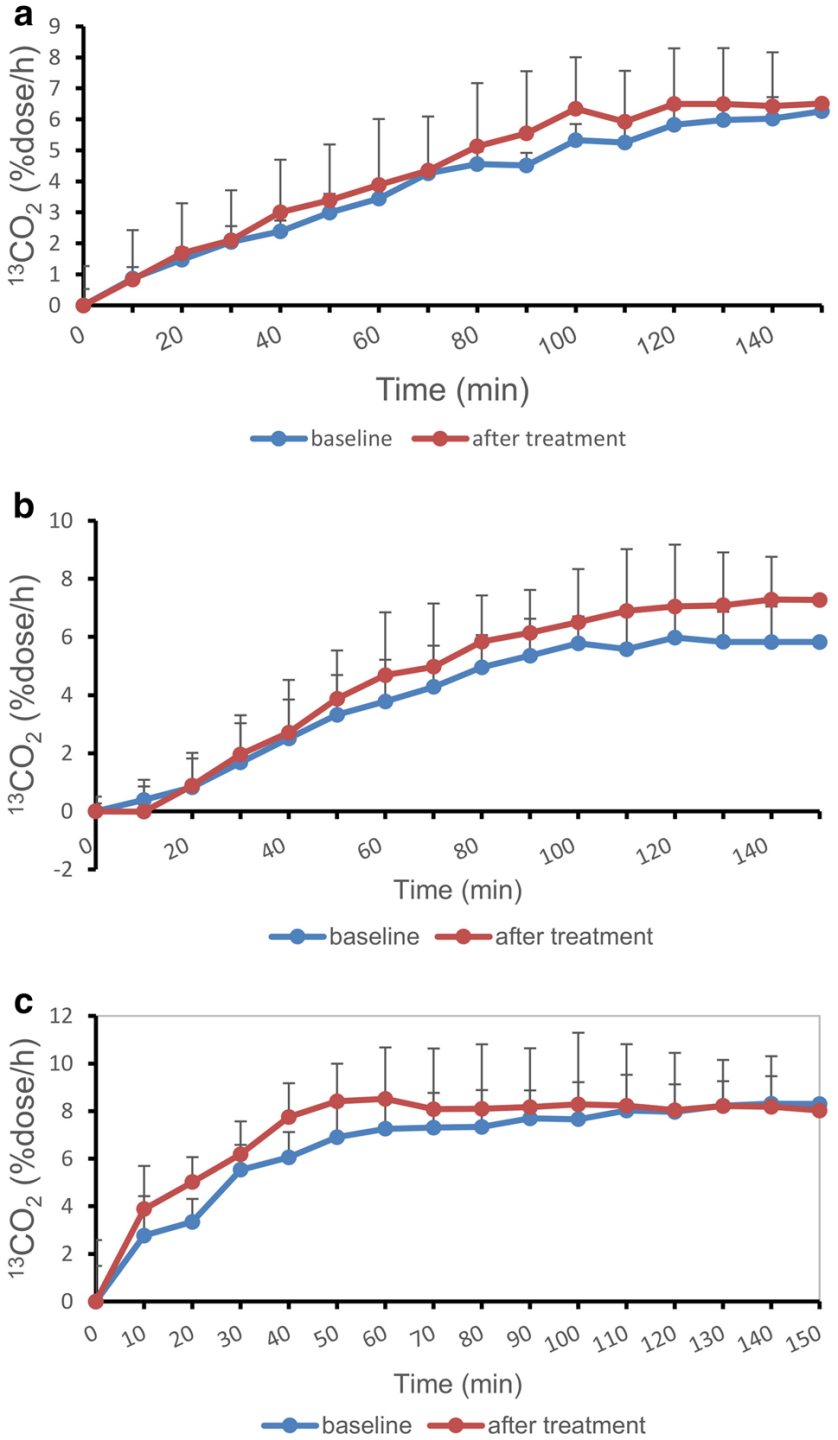
^13^CO_2_ excretion curves. **a** Comparison of orally administered [1-^13^C]glucose at baseline and after 2-week treatment with DPP-IV inhibitors. **b** Comparison of orally administered [2-^13^C]glucose at baseline and after 2-week treatment with DPP-IV inhibitors. **c** Comparison of orally administered [3-^13^C]glucose at baseline and after 2-week treatment with DPP-IV inhibitor

Follow-up breath tests were performed after 2-week treatment with DPP-IV inhibitors in five diabetic male (37–71 years, HbA1c 7.9 ± 1.9%). As shown in Table 7, AUC_150_ in all three breath tests increased after treatment, but the differences did not reach a statistical significance. An increase in ^13^CO_2_ levels after treatment was noted in late phase of [2-^13^C] and [1-^13^C]glucose breath test, whereas in early phase of [3-^13^C]glucose breath test (Fig. 5).

**Table 7:** AUC_150_ values of all breath tests in five diabetic patients before and after treatment with DPP-IV inhibitors

	Baseline	After treatment	*P*
[1-^13^C]glucose	576 ± 65	645 ± 246	NS
[2-^13^C]glucose	588 ± 160	695 ± 230	NS
[3-^13^C]glucose	972 ± 180	1071 ± 294	NS

Data are represented as mean [(%dose/h) min] ± SD

*NS* not significant

## Discussion

Breath testing has a major advantage over existing tests in that it can be performed non-invasively and repeatedly. Although glucose labeled with the stable isotope ^13^C has been used extensively for the diagnosis of diabetes, there are few studies using [2-^13^C] or [3-^13^C]glucose, which are too expensive to use clinically. [1-^13^C]glucose or uniformly labeled glucose has been used to evaluate glucose metabolism and insulin resistance as a non-invasive alternative [11, 12]. A particularly invasive method, hyperinsulinemic–euglycemic clamp testing, is considered to be the gold standard diagnostic test, but is unsuitable for routine clinical use. Although the oral glucose tolerance test is a commonly used procedure for the diagnoses of diabetes mellitus and intermediate stages of hyperglycemia, needle-associated fear is inevitable. This fear may possibly make a patient hesitate in getting a medical check-up at a hospital. Thus, non-invasive methods are clinically more desirable to evaluate patients at risk for diabetes mellitus.

Because of this, we previously reported a novel alternative method, the combined ^13^C-glucose breath test, to evaluate exogenous glucose metabolism [7]. Similar to this study, the increase in breath ^13^CO_2_ excretion was delayed in diabetic rats compared with the controls. The delayed increase in ^13^CO_2_ excretion could be attributed to impaired glucose uptake in diabetes, because all carbon molecules are metabolized after glucose enters the cells. Surprisingly enough, the utilization of [2-^13^C]glucose, but not [1-^13^C]glucose, is suppressed in the prediabetic stage. This result indicates that insulin resistance contributes to the decreased suppression of gluconeogenesis by insulin, which may slow the TCA cycle by removing oxaloacetate [10]. It has been suggested that the [2-^13^C]glucose breath test could be more useful to evaluate impaired glucose metabolism at an earlier stage. The differences in ^13^CO_2_ excretion between the [1-^13^C] and [2-^13^C]glucose breath tests are due to the different metabolic fates of each carbon molecule. The C2 carbon of glucose is converted to the C1 carbon of acetate, which then enters the TCA cycle, is oxidized, and is excreted as ^13^CO_2_ at the next turn of the TCA cycle. Unlike the C2 carbon, the C1 carbon of glucose enters the TCA cycle after conversion to the C2 carbon of acetate, yielding ^13^CO_2_ after the third and subsequent turns [6]. It is, therefore, supposed that the ^13^CO_2_ excretion curve after oral administration of [2-^13^C]glucose reflects a more rapid oxidation of glucose compared with [1-^13^C]glucose. The remaining C3 carbon atoms of glucose are oxidized and excreted as ^13^CO_2_ before entering the TCA cycle. Thus, since the metabolic fates of the various carbon atoms of glucose differ, combined ^13^C-glucose breath testing using [1-^13^C], [2-^13^C], and [3-^13^C]glucose could provide valuable information on the state of glucose metabolism.

If ^13^CO_2_ excretion is reduced in the [3-^13^C]glucose breath test, this results from impaired anaerobic glycolysis. If ^13^CO_2_ excretion is decreased after oral administration of [1-^13^C] or [2-^13^C]glucose, glucose oxidation through the TCA cycle would be impaired. When exogenous glucose is metabolized via gluconeogenesis, the C1 carbons cannot generate ^13^CO_2_. By contrast, half the amount of ^13^CO_2_ generated from the C2 carbons is detectable in expired breath samples [6]. Patients with increased ^13^CO_2_ excretion in the [2-^13^C]glucose breath test but reduced ^13^CO_2_ excretion in the [1-^13^C]glucose breath test have enhanced gluconeogenesis, but inhibited glucose oxidation. By contrast, patients with increased ^13^CO_2_ excretion on both [1-^13^C] and [2-^13^C]glucose breath tests have enhanced glucose oxidation. In patients with low ^13^CO_2_ excretion levels on all three breath tests, the glucose uptake by cells or all metabolic pathways including anaerobic glycolysis, gluconeogenesis, and oxidation must have been suppressed.

As shown in Tables 3 and 4, the ^13^CO_2_ excretion expressed as the AUC_150_ and *C*_max_ was significantly lower in diabetic patients after orally administering all the three types of labeled glucose. These results indicate that glucose metabolism is suppressed in diabetic patients. Hyperglycaemia has been reported to reduce glycogenolysis and gluconeogenesis in the liver but to enhance glycogen synthesis [11, 12], and these pathways are closely associated with glucose uptake by target tissues. Insulin resistance is characterized by the reduced ability of insulin to stimulate tissue uptake and insufficient glucose absorption, resulting in impaired glucose homeostasis [13]. These metabolic changes coincide with the reduced ^13^CO_2_ excretion on [3-^13^C]glucose breath testing (Fig. 1c). Among diabetic patients, although both *C*_max_ and AUC_150_ were significantly lower in the severe diabetes group after oral administration of [1-^13^C]glucose compared to the non-severe group, there were no significant differences between the two groups after oral administration of [3-^13^C] or [2-^13^C]glucose (Tables 5, 6). These results suggest that impaired sites of glucose metabolism differ widely among diabetic patients, and insufficient utilization of glucose in the TCA cycle may aggravate type 2 diabetes.

Type 2 diabetes is mainly characterized not only by insulin resistance, but also pancreatic β-cell dysfunction [14]. Other studies have also reported lower adenosine triphosphate (ATP) turnover rates in type 2 diabetes using ^31^P magnetic resonance spectroscopy [15, 16]. These changes can explain the lower ^13^CO_2_ excretion in the exhaled air in patients with advanced diabetes mellitus. Unexpectedly, as shown in Fig. 4, the ^13^CO_2_ excretion curves for the [2-^13^C] and [1-^13^C]glucose breath tests were quite well approximated, suggesting that the prompt utilization of the C2 carbon is more suppressed compared with C1 when small amounts of glucose are ingested orally. Thus, we would expect that the [2-^13^C]glucose breath test could be used clinically in the diagnosis of diabetes mellitus.

The delayed ^13^CO_2_ excretion and low AUC_150_ values in diabetic patients in the present study in humans coincide with the results of our previous animal study [7]. The utilization of [3-^13^C]glucose is enhanced just before the onset of diabetes, which indicates that the pathway is enhanced via glycolysis. By contrast, the results for the [1-^13^C]glucose breath tests did not change considerably in the animal model, suggesting that glucose oxidation would be impaired after the progression of diabetes mellitus. It is also possible that impaired glucose oxidation might contribute to the progression of diabetes, potentially leading to the chronic phase of diabetes. Thus, combined [1-^13^C] and [3-^13^C]glucose breath testing could distinguish between the reduced uptake and impaired oxidation of glucose.

The [2-^13^C]glucose breath test mainly reflects the prompt oxidation of glucose and/or gluconeogenesis. When exogenous glucose is metabolized via gluconeogenesis, the C1 position cannot generate ^13^CO_2_, while half of the ^13^CO_2_ generated from the C2 position appears in the expired breath samples [6, 17, 18]. Unlike the [1-^13^C]glucose breath test, the ^13^CO_2_ excretion curve in the [2-^13^C]glucose breath test is generated by both glucose oxidation and gluconeogenesis, because the C2 carbon entering the TCA cycle can be converted to ^13^CO_2_ during the second turn of the cycle. In other words, only the [2-^13^C]glucose breath test is able to evaluate gluconeogenesis. When exogenous glucose enters the TCA cycle, any ^13^CO_2_ obtained from [2-^13^C]glucose should appear more rapidly than the ^13^CO_2_ obtained from [1-^13^C]glucose. As shown in Fig. 4, the ^13^CO_2_ excretion curve obtained after oral administration of [2-^13^C]glucose was markedly more reduced than [1-^13^C]glucose, and the two curves are quite well approximated. This result suggests that a small amount of exogenous glucose may not be metabolized via gluconeogenesis at all, and rapid oxidation, as evaluated by the [2-^13^C]glucose breath test, would be suppressed more than slow oxidation, which is evaluated by the [1-^13^C]glucose breath test.

As compared with baseline, an increase of ^13^CO_2_ excretion was noted after treatment in all three breath tests. An increase in ^13^CO_2_ levels after treatment was noted in late phase of [2-^13^C] and [1-^13^C]glucose breath test, whereas in early phase of [3-^13^C]glucose breath test. These suggest that glucose metabolism, including absorption from the gut, the uptake of glucose, the anaerobic glycolysis system, and oxidation of glucose, should be improved as a whole. It is expected that longer treatment could achieve a better glucose metabolism.

The present study had several limitations. First, a small amount of glucose was used in the breath tests. The results might not be consistent when a greater quantity of glucose is orally administered. Second, the severity of the patients’ type 2 diabetes was evaluated only based on HbA1c. Additional studies will be necessary to compare the combined ^13^C-glucose breath tests with the glucose clamp technique, which is the gold standard for evaluating various aspects of glucose metabolism. Third, control subjects were significantly younger than diabetic patients. Although the metabolic change of glucose might downregulate with advancing age, follow-up breath tests demonstrated that ^13^CO_2_ excretion was increased after treatment and closed to that of normal controls. It is suggested that an effect of ageing on glucose breath tests would be limited. Finally, ^13^C-glucose is currently costly, and breath testing is time-consuming. Therefore, performing such time-consuming and costly testing is unreasonable as a screening technique for diabetes.

In conclusion, the [1-^13^C]glucose breath test, which has been used to evaluate glucose metabolism, is suitable for patients with late-stage diabetes, whereas the [2-^13^C]glucose breath test is ideal in the early stages. Although the [3-^13^C]glucose breath test is theoretically useful for evaluating the uptake of glucose and the anaerobic glycolysis system, it can be used in practice to distinguish reduced uptake from impaired oxidation of glucose in combination with the other two tests. To the best of our knowledge, this study is the first human study to evaluate exogenous glucose metabolism using [1-^13^C], [2-^13^C], and [3-^13^C]glucose breath tests in great detail. We would now like to develop combined glucose breath tests by extending our research to include diabetic patients with various etiologies of the disease, including endocrine disorders, steroid use, and insulin-dependent diabetes.
